# Navigating performance measures for ambulatory antimicrobial stewardship: a review of HEDIS® and other metrics the steward should know

**DOI:** 10.1017/ash.2024.468

**Published:** 2024-12-13

**Authors:** Christen J. Arena, Michael P. Veve, Steven T. Fried, Felisa Ware, Patricia Lee, Anita B. Shallal

**Affiliations:** 1 Department of Pharmacy, Henry Ford Hospital, Detroit, MI, USA; 2 Department of Pharmacy Practice, Eugene Applebaum College of Pharmacy and Health Sciences, Wayne State University, Detroit, MI, USA; 3 Department of Family Medicine, Henry Ford Health, Detroit, MI, USA; 4 Department of Payer Relations and Practice Transformation, Henry Ford Health, Detroit, MI, USA; 5 Department of Infectious Diseases, Henry Ford Hospital, Detroit, MI, USA

## Abstract

Ambulatory antimicrobial stewardship can be challenging due to disparities in resource allocation across the care continuum, competing priorities for ambulatory prescribers, ineffective communication strategies, and lack of incentive to prioritize antimicrobial stewardship program (ASP) initiatives. Efforts to monitor and compare outpatient antibiotic usage metrics have been implemented through quality measures (QM). Healthcare Effectiveness Data and Information Set (HEDIS®) represent standardized measures that examine the quality of antibiotic prescribing by region and across insurance health plans. Health systems with affiliated emergency departments and ambulatory clinics contribute patient data for HEDIS measure assessment and are directly related to value-based reimbursement, pay-for-performance, patient satisfaction measures, and payor incentives and rewards. There are four HEDIS® measures related to optimal antibiotic prescribing in upper respiratory tract diseases that ambulatory ASPs can leverage to develop and measure effective interventions while maintaining buy-in from providers: avoidance of antibiotic treatment for acute bronchitis/bronchiolitis, appropriate treatment for upper respiratory infection, appropriate testing for pharyngitis, and antibiotic utilization for respiratory conditions. Additionally, there are other QM assessed by the Centers for Medicare and Medicaid Services (CMS), including overuse of antibiotics for adult sinusitis. Ambulatory ASPs with limited resources should leverage HEDIS® to implement and measure successful interventions due to their pay-for-performance nature. The purpose of this review is to outline the HEDIS® measures related to infectious diseases in ambulatory care settings. This review also examines the barriers and enablers in ambulatory ASPs which play a crucial role in promoting responsible antibiotic use and the efforts to optimize patient outcomes.

## Introduction

In 2022, healthcare providers prescribed 236 million outpatient antibiotic prescriptions, where up to 50% are considered unnecessary.^1^ Ambulatory settings account for approximately 60% of all US antibiotic expenditures; unnecessary antibiotic use contributes to adverse drug events (ADEs) that results in >145 million emergency department (ED) visits annually.^2–4^ Despite the shift from hospital-based to ambulatory settings, most antimicrobial stewardship program (ASP) efforts remain within the hospital.^5^

In 2016, the Centers for Disease Control and Prevention (CDC) released the Core Elements of Outpatient Antibiotic Stewardship that encompass strategies for a successful ambulatory ASP.^6^ An important aspect to this framework is identifying high-priority infectious disease (ID) syndromes for intervention, especially scenarios where clinicians deviate from best practices or overutilize antibiotics.^6^ However, standards for diagnosis and treatment often differ between system-specific practice guidelines, national professional society guidelines, and real-world practice. Moreover, objective measures and metrics for performance evaluation are poorly developed with a paucity of data and resources for implementation to affect prescribing changes.^5^

An additional but important barrier to implementing ambulatory ASPs had been the lack of accountability through traditional regulatory bodies like Centers for Medicare and Medicaid Services (CMS).^3^ Only in the last decade had The Joint Commission issued requirements for ambulatory ASPs within health systems,^7^ followed by a condition of participation from CMS.^8^ This caused a shift from quantitative ASP measures, toward measures related to patient quality and safety, often tied to reimbursement and enhanced program value.^9^ Linking ambulatory ASPs to health system reimbursement is essential to the program’s outcomes.^5,6^

This review outlines ambulatory ASP performance measures (ie, HEDIS®) and other initiatives related to ID, examining barriers and enablers that influence responsible antibiotic use and efforts to optimize patient outcomes.

## The HEDIS® measures

### What is a HEDIS® measure?

Healthcare Effectiveness Data and Information Set (HEDIS®) is a standardized set of performance measures maintained by the National Committee for Quality Assurance (NCQA) used by >90% of health plans.^10,11^ CMS works with NCQA to collect HEDIS® measures (HM) from Medicare Special Need Plans, using this to assess quality of care delivered by health plans, track improvement, and focus efforts on identifying performance gaps to improve health care.^10^ HEDIS® includes >90 measures across 6 domains of care: (i) effectiveness of care, (ii) access/availability of care, (iii) experience of care, (iv) utilization and risk-adjusted utilization, v) health plan descriptive information, and (vi) measures reported using electronic clinical data systems.^11^ CMS offers “pay-for-performance incentives” or “value-based reimbursement” when insurers and providers aim to achieve compliance in all 6 domains.^10–12^

HM were first developed as a mechanism to compare managed care organization plan quality.^13^ HM create service accountability among health plans, providers, and medical institutions to justify the quality or value of healthcare plans, particularly when quality concerns related to underuse, overuse, or misuse of healthcare services and their associated patient harm arose during the late 1990s.^13^ Table 1 outlines the current HM developed for ID conditions, or three targeted clinical diagnoses that account for >30% of all outpatient antibiotic prescriptions: bronchitis, upper respiratory infections (URIs), and pharyngitis,^14^ along with additional CMS quality measures (QM). HM are reported in three age stratifications, where the total rate is the sum of the age stratifications: pediatrics (ie, 3 months–17 years), 18–64 years, and ≥65 years.

**Table 1.** A summary of quality measures, descriptions, and the antimicrobial stewardship target for performance measures

Measure Name	Abbreviation	Measure Description	Stewardship Target
Appropriate testing for pharyngitis	CWP (HEDIS®)	The percentage of episodes for members ≥3 years of age where the member was diagnosed as having pharyngitis, dispensed an antibiotic, and received a group A streptococcus (strep) test for the episode	Appropriate use of health care Efficiency and cost reduction Diagnostic stewardship Antibiotic selection and guideline concordance
Appropriate treatment for upper respiratory infection	URI (HEDIS®)	The percentage of episodes for members ≥3 months of age with a diagnosis of upper respiratory infection that did not result in an antibiotic dispensing event	Antibiotics are not indicated for diagnosis
Avoidance of antibiotic treatment for acute bronchitis/bronchiolitis	AAB (HEDIS®)	The percentage of episodes for members ≥3 months with a diagnosis of acute bronchitis/bronchiolitis that did not result in an antibiotic dispensing event	Antibiotics are not indicated for diagnosis
Antibiotic utilization for respiratory conditions*	AXR* (HEDIS®)	The percentage of episodes for members ≥3 months with a diagnosis of a respiratory condition that resulted in an antibiotic dispensing event	Prescription rates and duration of therapy for all acute respiratory illness diagnoses
Antibiotic prescribed for acute viral sinusitis	Quality ID #331 (CMS)	The percentage of patients, aged 18 years and older, with a diagnosis of acute viral sinusitis who were prescribed an antibiotic within 10 days after onset of symptoms	Antibiotics are not indicated for diagnosis
Appropriate choice of antibiotic: amoxicillin with or without clavulanate for acute bacterial sinusitis	Quality ID #332 (CMS)	The percentage of patients aged 18 years and older with a diagnosis	Appropriate use of health care Efficiency and cost reduction Antibiotic selection and guideline concordance

* This measure is designed to capture the frequency of antibiotic utilization for respiratory conditions and is meant to be used for internal evaluation only.

HM are calculated using outpatient and ED patient-level data through International Classification of Diseases, 10^th^ edition (ICD-10) for disease state diagnosis with an eligible denominator during the evaluation period, as derived from the affiliated health system. HM competency and assessment of progress can be challenging to interpret and rely on competitor payor and national data to set thresholds. Ambulatory ASPs should develop a strong relationship with the affiliated health system’s payor relations leadership for an individualized approach to HM progress and goals.

### Measure #1—avoidance of antibiotic treatment for acute bronchitis/bronchiolitis (AAB)

The AAB measure evaluates the percentage of patients ≥3 months of age with an outpatient or ED visit diagnosed as having acute bronchitis/bronchiolitis (ICD-10 J20.0–J20.9) without select comorbid conditions who did not receive an antibiotic prescription within 3 days of the encounter.^14^ Patients with documented medical reasons for prescribing or dispensing antibiotics, or who used hospice services during the evaluation period, are excluded. Higher percentages indicate better care and adherence to evidence-based guidelines. The goal is to avoid unnecessary antibiotic use to minimize patient medication ADEs, reduce health expenditure, and combat antibiotic resistance.

Acute bronchitis is a top ten reason for outpatient visits in the United States,^15,16^ with 70% resulting in unnecessary antibiotic prescriptions due to viral etiology.^9^ Thus, it is a major stewardship target for independent and federal agencies.^1,17^ Multiple national associations have published best practice recommendations to avoid antibiotics in URI, suggesting supportive care and symptom management as the mainstay of treatment.^18^

A 2023 study evaluated an effective ambulatory ASP intervention in urgent care, targeting reduced antibiotic prescribing for respiratory illnesses, including bronchitis.^16^ The stewardship intervention included clinician and patient education, electronic health record (EHR) tools, clinician benchmarking dashboard, incentivized performance with financial support, and media.^16^ Three-month post-intervention, there was a 47% reduction in antibiotic prescribing (OR, 0.53; 95%CI, 0.44–0.63; *P* <0.001).^16^ A multifaceted approach which focused on active clinician- and patient-focused educational materials was successful in reducing prescribing rates for bronchitis by 10.1% in the intervention period.^19^ Another study included various outpatient settings who implemented a passive, prescriber-directed best practice advisory, and optional education regarding acute bronchitis treatment; antibiotic prescribing rates decreased by 9.4% (*P* <0.001) post-intervention.^20^

### Measure #2 - appropriate treatment for URI

The URI measure evaluates the percentage of patients aged ≥3 months with an outpatient or ED visit with a non-bronchitis URI diagnosis (ICD-10 J00, J06.0, J06.9) who did not receive an antibiotic prescription <3 days of the encounter.^11^ Encounters with competing diagnosis are excluded from the measure. Like the AAB measure, the URI measure aims to maximize the percentage of episodes managed without antibiotics.^11^

Although mostly viral, URIs lead to antibiotic prescriptions in up to 32% of cases.^21^ As such, national efforts have been designed to target inappropriate antibiotic prescribing for URI. National societies have disseminated strategies for treatment,^22^ with recommendations based on the meta-analysis of 15 randomized controlled trials reporting increased patient ADEs when treated with antibiotics, thus supporting the recommendation against antibiotic therapy.^23^ Promoting over-the-counter symptomatic relief is a first-line recommendation, with low minor ADEs and proven to shorten illness duration.^24^ Effective and impactful strategies to curb inappropriate antibiotic prescribing in URIs have been published, including a multifaceted intervention that reduced inappropriate URI prescriptions in outpatient pediatric and adult patients.^25^ Patient and provider educational materials along with a computer-based dashboard for provider URI prescribing reduced antibiotic prescriptions from 41% pre-intervention to 33% during the intervention.^25^ Another successful ASP intervention was conducted in rural China, reducing antibiotic prescribing in URIs for pediatric outpatients though provider training, guidelines, peer-review meetings, and caregiver education over 6 months.^26^ The authors concluded a 29% absolute risk reduction in antibiotic prescribing due to the intervention (95%CI, –42 to –16; *P* = 0.002).^26^ Despite these positive findings,^25–27^ studies evaluating education, training, or tools used for patient or provider-directed URI interventions have shown heterogenous results.^28^ Separate studies highlight inconsistencies in antibiotic prescribing when comparing the use of educational materials to control groups.^28,29^

A potentially underutilized method to reduce antibiotic use is delayed antibiotic prescription-filling interventions with various approaches: delayed prescriptions, patient-led prescriptions, post-dated prescriptions, delayed collection, and delayed re-contact.^28^ Literature on delayed antibiotic prescribing (ie, “wait and watch”) showed efficacy as a stewardship intervention, as patients who received delayed antibiotics were less likely to use antibiotics compared to the group who received a prescription at the encounter.^28^ Delayed antibiotic interventions resulted in similar patient satisfaction and fewer antibiotic ADEs (specifically, a lower rate of diarrhea) between groups.^28^

### Measure #3—appropriate testing for pharyngitis (CWP)

CWP assesses the percentage of patients aged ≥3 years with an outpatient or ED visit diagnosis of pharyngitis (ICD-10 J02.8, J02.9, J02.0) who had an appropriate antibiotic ordered and received a group A streptococcus (GAS) diagnostic test within 3 days of the encounter.^11^ Patients receiving hospice services during the encounter or who received antibiotics <30 days of the encounter are excluded. The purpose of this measure is to reduce unnecessary antibiotic use by confirming GAS diagnosis via testing prior to antibiotic prescription.

Pharyngitis, with either viral or bacterial etiologies, is another leading cause of outpatient visits. Unlike other URIs, rapid antigen detection tests (RADT) distinguish between viral and GAS pharyngitis within minutes, therefore optimizing antibiotics through a test-and-treat method. Patients who undergo RADT also demonstrate a higher level of adherence to the test-and-treat approach.^11^ Despite the CWP measure and the high sensitivity of RADT for GAS pharyngitis, appropriate testing rates have declined nationwide across all insurance plans recently.^11^ One study found that antibiotics are prescribed in 70% of pediatric primary care visits with unconfirmed pharyngitis.^30^

When differentiating between bacterial and viral pharyngitis, “clinical diagnosis cannot be made with certainty even by the most experienced physicians.”^31,32^ Bacterial pharyngitis causes an estimated 30% and 15% of pharyngitis episodes in children and adults, respectively,^33^ and viruses are the most common cause of pharyngitis across all age groups. Prediction tools for identifying GAS pharyngitis based on clinical features have been published, but fail to demonstrate diagnostic accuracy, particularly in children.^30^ This underscores the critical importance of conducting diagnostic tests prior to prescribing antibiotics. The reluctance to not prescribe antibiotics for pharyngitis may stem from the potential risks of untreated disease complications,^32^ but adherence of this measure can mitigate antibiotic overuse.

With high specificity of RADT and cultures, why do physicians still rely more on their clinical judgment when it has been described as faulty?^34^ Avent et al discussed the need for a sustainable “top-down strategy” in the ambulatory setting.^34^ HAPPY AUDIT was a multinational, pre-post-study evaluating a multifaceted intervention targeting general practitioners’ treatment of patients with respiratory infections, including GAS pharyngitis, and was effective for reducing the number of antibiotic prescriptions in six countries with a sustainable intervention after a 6-year audit.^35–37^ Molero et al evaluated the practitioners who participated in the HAPPY AUDIT intervention in Spain, inviting them to participate in another pre- post-intervention and 6-year audit with a focus on acute pharyngitis RADT and antibiotic prescribing.^37^ Regrettably, the intervention’s sustainability was suboptimal: RADTs were utilized less (51.7% to 49.4%), and increased antibiotic prescriptions (21.3%–36.1%, *P* <0.001) resulted in over 2-fold increase in antibiotics prescribed after 6 years (odds ratio: 2.24, 95% confidence interval: 1.73–2.89).^37^

### Measure #4—antibiotic utilization for respiratory conditions (AXR)

Of the four measures, the most recently published HM was AXR in 2022. The purpose of this measure is to summarize data on the percentage of outpatient episodes (i.e., telephone encounter, ED visit, e-visit, virtual check-in) for members > 3 months of age with a diagnosis of a respiratory condition that resulted in an antibiotic dispensing event.^11^ This measure coincides with the three previously discussed measures, but with less variability in diagnosis and coding practices for health plans to compare prescribing more accurately for these respiratory conditions.^38^ Since NCQA does not view higher or lower service counts as indicative of better or worse performance for this measure, organizations can only leverage this measure for internal benchmarks and evaluation.

### Additional QM

CMS has also developed QM with a goal to deliver safe, efficient, and equitable patient-centered care. QM are important for public reporting and pay-for-reporting programs.^39^ As of 2024, two ASP-related QM fall under the merit-based incentive payment systems, including avoiding overuse of antibiotic prescribing in adult sinusitis and prescribing appropriate antibiotics for acute bacterial sinusitis.^40,41^ Measures are met when antibiotic prescribing occurs <10 days after sinusitis symptoms onset, and when amoxicillin (with/without clavulanate) was prescribed as the first-line antibiotic at diagnosis. A systematic review found no clear benefits of antibiotics over placebo (or no treatment) for rapid recovery in adults with acute rhinosinusitis in the ambulatory setting but noted an increase in side effects.^42^ Like other QM, bundled stewardship interventions were associated with guideline-concordant antibiotic use for sinusitis including telemedicine visits.^43^ These additional measures are outlined in Table 1.

Notably, there are additional QM tied to patient immunizations among the general population and people living with HIV that may be worth considering based on the ASP’s capacity.^44^ Focusing on preventative measures, including vaccinations for pneumococcal disease, SARS-CoV-2, and influenza, can serve as a proactive stewardship strategy that can reduce the incidence of respiratory illnesses. This, in turn, could decrease the number of healthcare episodes for such conditions, thereby minimizing the likelihood of unnecessary antibiotic prescriptions.

### How should the ASP leverage HEDIS® and other performance measures?

**Figure 1.**
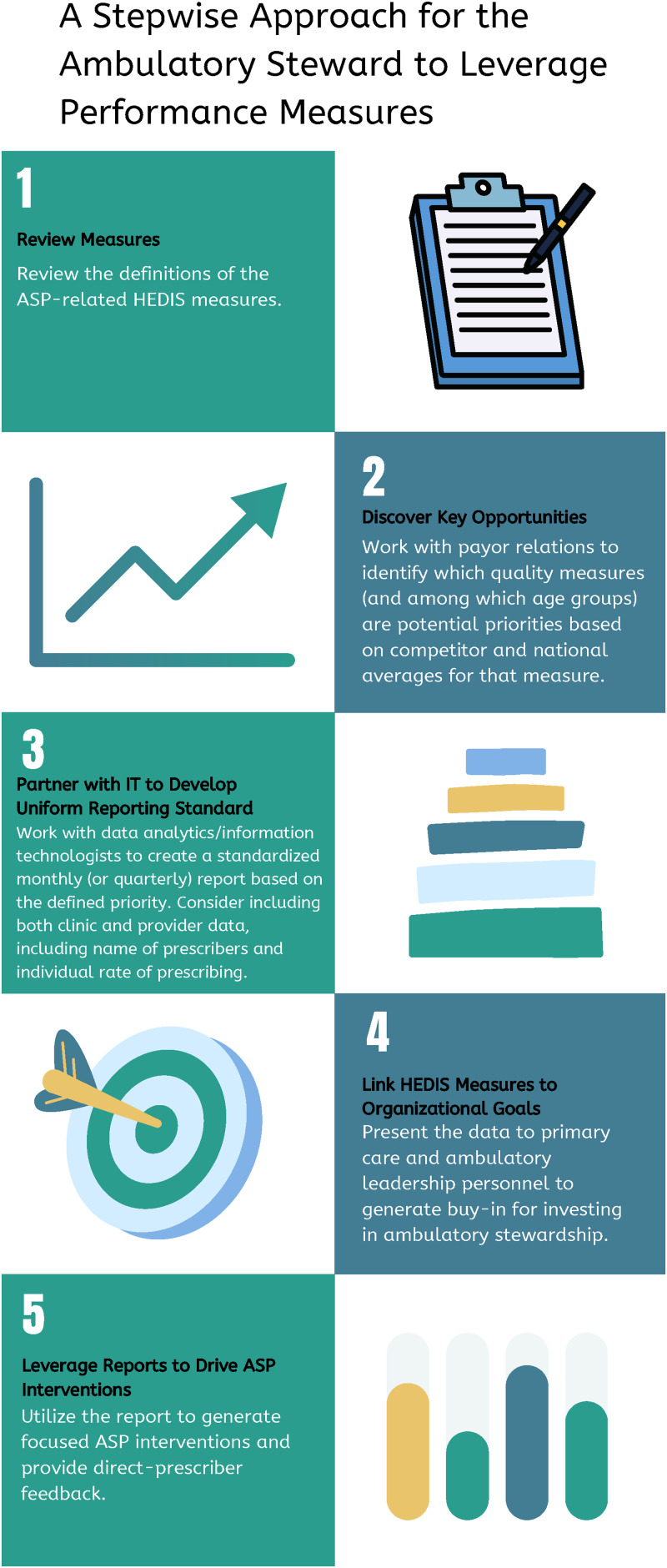
A stepwise approach for the ambulatory steward to leverage the performance measures. Source: https://www.canva.com/design/DAGRspmkee0/8wJiv1McJECYl2EayQYL6w/edit

Newly established ambulatory ASPs may lack strategic direction or struggle to identify high-value targets for intervention. The ASP’s initial stewardship targets should include HM given their implications on value-based reimbursement, pay-for-performance metrics, patient satisfaction, and incentivization to participate in other performance measures or rewards. The ambulatory ASP should gather baseline and trended HEDIS® data to identify pragmatic interventions and use these insights to strengthen provider relationships for future initiatives beyond HEDIS®. Methods to disseminate constructive feedback to prescribers related to HM performance could include e-mail, through the EHR public dashboard display, or through routine presentation at stakeholder meetings. Figure 1 proposes a stepwise approach. Each institution should formalize specific goals with stakeholders (ie, organizational leadership) based on HEDIS benchmarks as key performance indicators for ambulatory practices and providers that can be tied to financial incentives and disincentives, as well as performance reviews. Ideally, this information should be presented to end users and leadership through easy-to-interpret displays and in other data-sharing avenues (ie, personalized report cards, dashboards).

### A review of literature describing barriers and challenges

#### Understanding the measures

A major challenge for ambulatory ASP lies in the complexity of understanding performance measurements as both an assessment of effectiveness and a mechanism to drive change. Reported barriers to embracing HM include lack of understanding clinical relevance of measures, the idea that prescribing and health outcomes are probabilistic, the inability to juggle competing priorities, the complexity of insurance health plans and HM benefits, and how HM are calculated and compared.^13^ Some clinicians may challenge that the use of standardized measures is problematic in capturing appropriateness through the context of individualized patient care and could critique HM as a moving target that lacks a clearly defined goal.^13^ Ambulatory ASPs should work to educate providers on HEDIS® or other QM to ensure transparency, education, and constructive feedback for all prescribers.

#### Provider factors

With increasing emphasis in avoiding hospitalization admissions, outpatient healthcare providers face many competing priorities, including many other important non-infectious HM. Time constraints, high patient volumes, and decision-making fatigue throughout the clinic day are linked to increased antibiotic prescribing.^45,46^ In addition, providers often report concerns about perceived patient demand, or patient/caregiver satisfaction if they do not prescribe antibiotics.^20^ Providers may believe that their prescribing behavior does not contribute to antibiotic overuse, attributing it instead to other providers in different settings.^47^ Default options in EHRs may also influence treatment decisions regarding antibiotic choice or duration, which may not align with current guidelines. Some data have described a significant difference in prescribing between physicians and advanced practice providers (APPs), where odds of prescription were 30% higher when APPs were part of the visit.^48^ Variations in provider specialty can all contribute to diagnostic uncertainty, which may increase the prescription of antibiotics.^49^ Additionally, there is growing evidence that prescriptions for antibiotics lack equity and often differ based on race, ethnicity, and language.^50^ These social, behavioral, and contextual factors contribute to inappropriate antimicrobial prescribing, affecting ambulatory ASP.

### Enablers and opportunities

#### Reflection of contemporary practice

Unfortunately, health systems’ priorities do not always align with the areas of major deficiencies in clinical practice. For example, acute otitis media (AOM) accounts for 8.7 million antimicrobial prescriptions annually, even though for most children in high-income countries with mild AOM, the infection spontaneously remits without antimicrobials.^51,52^ Not only are antimicrobials frequently inappropriately prescribed, but in a study of 926 children diagnosed as having AOM in the United States, the duration of therapy was >5 days and ≥10 days in 94% and 55% of participants, respectively.^53^ Like inpatient practice, the issue is not only with unnecessary antimicrobial prescriptions or prolonged durations, but also inappropriate agent selection. Although there are clear guidelines regarding “watchful waiting” and first-line treatment for acute rhinosinusitis, less effective, non-first-line agents like macrolides are used up to 60% of the time.^54,55^ Finally, although urinary tract infections (UTI) account for over 8.6 million ambulatory care visits per year, the rate of treatment for asymptomatic bacteriuria (ASB), particularly among elderly patients or those residing in long-term care facilities, remains largely unexplored.^56^ Despite the clear evidence that AOM, sinusitis, and ASB represent an area requiring ASP interventions,^55,57,58^ these disease states are not tracked for HM reimbursement. Additionally, there exists no metric that targets antimicrobial therapy duration, a known independent risk factor for *C. difficile* infection.^59^

#### Organization support and resources

National and governmental organizations are supporting ambulatory ASP efforts more than ever, in addition to inpatient initiatives. The CDC recently published guidance to improve outpatient antibiotic prescribing, identify targets for ASP interventions, and measure and evaluate performance and progress overtime.^60^ An example from this guidance looks at tracking excess antibiotic prescription duration, containing listed data requirements to be able to track durations, such as EHR pharmacy data, and followed by a bulleted list of advantages and disadvantages for tracking excess antibiotic prescription durations. HM for outpatient antibiotic prescribing are also broken down each by measure, description, and a defined numerator and denominator.^60^ An overwhelming part of tracking antibiotic use, specifically in the ambulatory setting, is the need for technical resources. Fortunately, there are several helpful resources to understand where to start: “CDC Outpatient Treatment Recommendations,” “HEDIS® Measures” website, “MITIGATE AMS Toolkit,” and “Implementation Guide for Ambulatory Care Antibiotic Stewardship.”^60^ There is significant potential to enhance data quality control and optimize analyst time. Under-resourced institutions often face challenges in extracting the necessary data elements for evaluating HM. However, as health systems increasingly adopt electronic clinical QM, this shift may alleviate some of the burdens and address the inequitable opportunities associated with pay-for-performance incentives.

#### Perks of consolidated health systems for ambulatory ASP

To support stewardship expansion, an ambulatory ASP program must include diversity in healthcare specialties, necessary resources and support, and a centralized approach to infrastructure that healthcare systems themselves can provide as a key stakeholder.^61^ Rodzik et al describes the trend in consolidated healthcare delivery in the United States, with affiliation of 72% of hospitals and 49% primary care physicians with health systems as of 2018.^61^ An advantage of a centralized approach is the ability to use simplified, standardized, system-wide institutional guidelines/policies, promoting benchmarks for performance standards that can improve on the HM. Standardization is key to facilitating patients receiving a more harmonized antibiotic guidance, along with optimal treatment strategies.^61^ Considering these factors, health systems actively pursue Joint Commission accreditation to meet or exceed patient care and safety standards, while private outpatient settings may choose not to participate.^60^

#### Legislative change, comparative scorecards, and multi-institutional efforts

**Figure 2.**
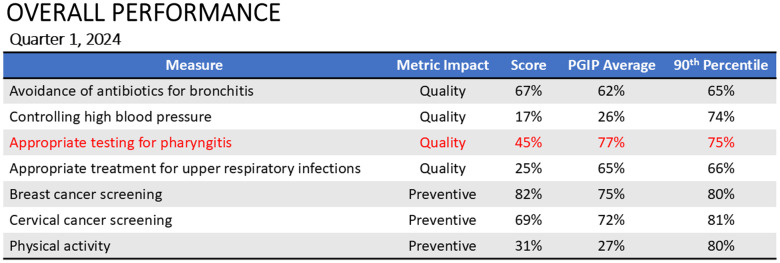
Example of a HEDIS® measure scorecard to inform opportunities for improvement. In this example, the institution is scoring significantly lower than both the Physician Group Incentive Program (PGIP) average and the 90^th^ percentile in the measure of appropriate testing for pharyngitis. For the antimicrobial steward reviewing this scorecard, it would be important to prioritize that measure over avoiding antibiotics for bronchitis and appropriate treatment for upper respiratory infections, where the program is scoring better than the average.

Advocacy is essential to informing policy around ambulatory infectious syndromes and translate it into actionable change and quantifiable results.^6^ Health systems should collaborate with other hospitals on quality initiatives to improve the delivery of quality patient care. For example, the Michigan Hospital Medicine Safety Consortium (HMS) unites hospitals statewide to collect and analyze data, implement improvement strategies, and evaluate change over time.^62^ Although HMS currently focuses on hospitalized patients, the development of validated measures of inappropriate diagnosis for UTI and community acquired pneumonia has reduced antibiotic use, including for ASB.^63,64^ These measures are now endorsed by the National Quality Forum. Sharing data between institutions can also create comparative scorecards whereby ambulatory ASP can select which measures are top priority to focus efforts to incentivize and generate buy-in with the C-suite (Figure 2).

#### AI and predictive algorithms to improve antibiotic use

The importance of leveraging the EHR in the ambulatory care setting has been made evident, specifically in chronic disease state management.^65,66^ The EHR domain represents a “cultural revolution” with inherent challenges but unbounded prospects.^67^ Marra et al discuss how artificial intelligence (AI) can individualize treatment in ASP using real-time algorithms based on patient antimicrobial history.^68^ Integration of advanced microbiology laboratory instrumentation with AI can enhance the speed and accuracy of predicting antimicrobial resistance patterns.^68,69^ Prioritizing change to enhance patient care using contemporary tools and standardized methods is paramount.

## Conclusion

Leveraging HM can help ambulatory ASPs standardize performance expectations, secure institutional support, and set appropriate benchmarking. This approach can incentivize responsible antibiotic use, optimize patient outcomes, and provide a framework for developing future interventions.
